# Operation in Simple Fractures

**Published:** 1900-06-02

**Authors:** 


### OPERATION IN SIMPLE FRACTURES.

Lecturing at Guy's Hospital, the other day, Mr. Arbuthnot Lane reviewed the treatment of simple fractures, and urged again and with still greater force what he has often pointed out before, that it is in many cases quite impossible to restore a broken bone to its proper position without laying it bare. He has now also shown that the consequences of malposition after fracture are far more serious than some have imagined, including as they do various changes in the neighbouring joints set up not directly by the injury, but as the result of abnormal mechanical strain arising from the altered form of the ill-united bone. By comparing the lines of treatment recommended by various authorities with the reasons given by some of them for their teachings, he has also shown how great is the confusion in the minds of many surgeons in regard to the mechanism involved, both in the production and the reduction of fractures. He shows also that surgeons who hold very similar, and as he believes perfectly incorrect views as to the mechanical factors that oppose the reduction and retention of the fragments of a broken bone in accurate apposition, yet appear from their accounts to obtain equally good results by methods which to the ordinary mind are mechanically absolutely antagonistic both in practice and in principle. This lecture is to all intents and purpose a challenge to surgeons to show their results, and to prove them by the aid of the *x* rays. "Those who continue to assert that they are able to restore broken bones to their original form can readily substantiate the truth of their statements by means of the radiograph, and if they have any confidence in them will doubtless do so. Up to the present, however," he says, "I have seen no evidence of this kind brought forward, but merely vague statements that could not be met." Mr. Arbuthnot Lane insists, then, that in every simple fracture in which it is important to the individual that the original form of his skeleton shall be retained, and his mechanics suffer no alteration, the surgeon should, failing to obtain accurate apposition as determined by the radiograph, cut down on the seat of fracture and restore the bone or bones to their original form. He must not be satisfied with what is clinically called a "good position," when by operation he can obtain a perfect result, and in the case of the leg this is particularly important. This challenge and this teaching will, we think, give rise to considerable controversy. On the one hand, it is clear that the specialist with his tools about him, and with all the advantages of hospital surroundings at his disposal, can obtain results by operation which are probably unattainable, so far as mechanical perfection is concerned, by any other means. But it does not follow that surgeons less favourably placed would be justified in following his teaching.

